# Repetitive Transcranial Electrical Stimulation Induces Quantified Changes in Resting Cerebral Perfusion Measured from Arterial Spin Labeling

**DOI:** 10.1155/2018/5769861

**Published:** 2018-09-05

**Authors:** Matthew S. Sherwood, Aaron T. Madaris, Casserly R. Mullenger, R. Andy McKinley

**Affiliations:** ^1^Infoscitex, a DCS company, 4027 Colonel Glenn Hwy, Beavercreek, OH 45431, USA; ^2^Department of Biomedical, Industrial & Human Factors Engineering, Wright State University, 3640 Colonel Glenn Hwy, Dayton, OH 45435, USA; ^3^Air Force Research Laboratory, U.S. Air Force, 2510 Fifth Street, Bldg 840, Wright-Patterson AFB, OH 45433-7951, USA

## Abstract

The use of transcranial electrical stimulation (TES) as a method to augment neural activity has increased in popularity in the last decade and a half. The specific application of TES to the left prefrontal cortex has been shown to produce broad cognitive effects; however, the neural mechanisms underlying these effects remain unknown. In this work, we evaluated the effect of repetitive TES on cerebral perfusion. Stimulation was applied to the left prefrontal cortex on three consecutive days, and resting cerebral perfusion was quantified before and after stimulation using arterial spin labeling. Perfusion was found to decrease significantly more in a matched sham stimulation group than in a group receiving active stimulation across many areas of the brain. These changes were found to originate in the locus coeruleus and were broadly distributed in the neocortex. The changes in the neocortex may be a direct result of the stimulation or an indirect result via the changes in the noradrenergic system produced from the altered activity of the locus coeruleus. These findings indicate that anodal left prefrontal stimulation alters the activity of the locus coeruleus, and this altered activity may excite the noradrenergic system producing the broad behavioral effects that have been reported.

## 1. Introduction (AM)

Transcranial electrical stimulation (TES) has experienced increased interest over the last 15 years [1]. The application of TES using a weak, constant current delivered to the scalp is referred to as transcranial direct current stimulation (tDCS) [2]. This method has been presented by many groups as a feasible process for stimulation of the brain to augment neural activity [3–7]. The specific application of tDCS with the anode placed over the left prefrontal cortex has been routinely applied in the literature with demonstrable behavioral effects in combating performance decrements associated with vigilance [8], decreasing the effect of fatigue on cognitive performance [9, 10], accelerating learning processes [2, 3, 11, 12], enhancing multitasking performance [13], and improving procedural memory [14].

Clark et al. [11] implemented 2 mA anodal left prefrontal tDCS while performing a task involving the identification of threat-related objects in a naturalistic environment. Using dynamic Bayesian network analysis, they indicated that the right frontal and parietal cortices were involved in the learning processes of their identification task. Furthermore, they reported that the group receiving full-current (2 mA) tDCS performed significantly better than the one that received low-current (0.1 mA) stimulation. McKinley et al. [12] provided support for the findings of Clark and colleagues using a realistic visual search task implanted with synthetic aperture radar images. They stated that participants who received anodal left prefrontal tDCS attained enhanced visual search accuracies compared to those supplied with sham or no stimulation. Effects of anodal left prefrontal tDCS have also been observed in decreasing the effects of fatigue on cognitive performance. Using a cohort of 30 participants (10 placebo gum, 10 caffeine gum with sham tDCS, and 10 2 mA anodal left prefrontal tDCS with placebo gum), McIntire et al. [9] performed psychomotor vigilance tasks, delayed matching-to-sample working memory tasks, and the Mackworth clock test throughout 30 hours of continuous wakefulness. They reported improved latencies in working memory tasks and faster reaction times in psychomotor tasks in the groups receiving active tDCS and caffeine gum compared to placebo throughout the sleep deprivation period. Altogether, these findings provide evidence for the central role of the prefrontal cortex in vigilance, accelerated learning, fatigue, and multitasking performance but indicate that tDCS may be utilized to maintain performance levels in environments requiring little to no rest or settings required sustained attentional focus.

Despite the broad applications of tDCS and those specific to anodal left prefrontal stimulation, the neural mechanisms underlying tDCS are not well understood. It has been suggested that anodal tDCS increases excitability in the neocortex [6] by altering neuronal membrane potentials [15]. This theory is supported by findings of enhanced glutamatergic activity following the application of anodal tDCS [2]. Neuroplasticity, the ability of the brain to form and restructure synaptic connections [16], is thought to coincide with increased glutamatergic activity [2] as evidenced in the lasting behavioral effects from tDCS (e.g., [9, 10]) and the acceleration of learning processes [3, 11, 12]. However, recent evidence suggests that the neuroplastic effects of tDCS have some dependence on synaptic activity during stimulation [7].

A growing method for studying neural processes is through the measurement of cerebral perfusion. The *in vivo* quantification of cerebral perfusion (referred to as cerebral blood flow (CBF) mL/100 mg/min) can be performed noninvasively using magnetic resonance imaging (MRI) through an arterial spin labeling (ASL) pulse sequence [17, 18]. ASL is a clinical method that has been used to identify early pathophysiological changes in Alzheimer's disease and other disorders such as dementia [18, 19]. In comparison to signals based on blood oxygen, CBF has better reliability and intersubject variability [20]. Furthermore, CBF is directly responsible for the delivery of glucose and oxygen. Both oxygen and glucose are necessary to maintain adenosine triphosphate (ATP) production and need to be replenished to support continued neural activity. Although CBF is not a direct measure of neural activity, it is a tightly coupled correlate: CBF changes with neural activity such as that which occurs during task activation or with changing metabolism [21]. Evidence published just this year indicates that this coupling is electrical: extracellular K^+^ activates capillary endothelial cells which then signal upstream arteriolar dilation [22]. The extracellular concentration of K^+^ increases during neural activity, thereby signaling enhanced vasodilation and increased blood flow to the supporting capillary bed.

The study of resting CBF in anodal left prefrontal tDCS may provide critical insights as increased glutamatergic activity associated with anodal tDCS would manifest as enhanced cerebral perfusion. Few previous studies have utilized ASL to assess the neural effects of tDCS. In some studies, anodal tDCS led increased regional CBF in the brain tissue underneath the stimulation site, with reliable and reproducible results within and between subjects. Furthermore, transfer effects were observed in brain regions functionally connected to the stimulation site [7]. Importantly, immediate and lasting changes in CBF have been associated with anodal left prefrontal tDCS [23]. The goal of this study is to enhance our understanding of the underlying neural mechanisms associated with repetitive anodal tDCS to the left prefrontal cortex through the study of resting cerebral perfusion. The study consisted of anodal left prefrontal tDCS applied on three consecutive days with the same procedures performed on each day to assess the additive effects of tDCS. In this work, we present preliminary findings of a larger, ongoing study.

## 2. Materials and Methods

### 2.1. Participants

A total of 28 healthy, active duty, Air Force military members recruited from Wright-Patterson Air Force Base volunteered to participate in this study. Participants were excluded from participation if they had any neurological or psychological diagnoses; vision, hearing, or motor control impairments; or recent trauma or hospitalization. Participants were also excluded if they currently took any medication which may affect cognitive function or if they were dependent on alcohol, caffeine, or nicotine. Written informed consent was obtained from each participant prior to any experimental procedures which were approved by the Air Force Research Laboratory Institutional Review Board at Wright-Patterson Air Force Base under Protocol number FWR20130126H. Participants eligible for compensation (i.e., if participation occurred in an off-duty status) received equal remuneration. Of the 28 participants recruited, eight were excluded due to medical disqualification (*n* = 2), incomplete data or corrupted data (*n* = 2), or failure to complete all three sessions in three consecutive days (*n* = 4).

Participants were randomly assigned to one of two groups. Both groups received the same instructions and performed the same tasks with the exception of the stimulation that was received. In the experimental group (ACT, *n* = 11, mean age = 24.5 ± 2.6), 2 mA stimulation was provided for 30 minutes while in the control group (CON, *n* = 9, mean age = 25.9 ± 3.2) sham stimulation consisting of 2 mA stimulation for 30 s. Participants in each group were blinded to the validity of the simulation (i.e., not aware of the stimulation condition) and naïve to TES (i.e., first time receiving TES).

All participants completed three experimental sessions on three consecutive days. Each session was separated by 24 hours. The sessions were conducted in the evening so as to not conflict with the working day but also due to the MRI availability. Participants completed the experimental sessions in groups of two with staggered start times (see Table 1). Start times were held consistent across the three sessions and were counterbalanced across groups.

### 2.2. Transcranial DC Stimulation

On each of the three sessions, anodal stimulation was applied to the left prefrontal cortex (approximately F3) with the cathode placed on the contralateral bicep. During stimulation, participants completed a 30 min laboratory vigilance task [24]. The electric stimulation (MagStim DC Stimulator, Magstim Company Limited, Whitland, UK) delivered a constant 2 mA through a ring of five custom Na/NaCl electrodes. The electrodes were arranged in a 1.6 cm radius circle and separated by 0.1 cm (outer edge to outer edge). The same ring configuration was used at the cathode location. The 2 mA stimulation was distributed evenly among the five electrodes. The stimulator is battery-powered and utilizes multistage current monitoring to ensure constant current levels are delivered to the anode. Each electrode was placed in a small “cup” and secured to the participant using medical bandages. The electrode cups were filled with highly conductive gel (SignaGel, Parker Laboratories, Fairfield, NJ) to ensure current transfer to the scalp.

### 2.3. MRI Acquisition

At each session, MRI data was acquired prior to and approximately 0.5 hours following the application of tDCS. The MRI acquisition consisted of the following sequences: a 12 min resting-state functional MRI (fMRI), three 10 min task fMRIs, T1-weighted MRI, diffusion tensor imaging (DTI), magnetic resonance spectroscopy (MRS), and resting ASL. As this work is part of a larger, ongoing study, we will only be presenting the resting ASL data in this work. However, it is important to discuss the three task fMRIs where participants completed a dual (verbal and spatial) *n*-back task. This task was conducted in a boxcar design with 48 s control and task blocks, each with 16–3 s trials. During each trial, a letter was displayed on a 3 × 3 grid for 500 ms. Participants were asked to provide one response if the current letter was the same as the *n*th previous letter that was presented and another response if the current letter was in the same position on a 3 × 3 grid as the *n*th previous letter. For control blocks, the letter was replaced with a dot and participants were instructed to provide one response if the dot was on the right side of the grid and another for the left. For the first run, *n* was set to 2. *n* for the second run was determined from the performance of the first run (if performance was 90 or above, *n* incremented; if less than 70, *n* decremented; otherwise, *n* remained the same) and the third run from the second.

Structural (T1-weighted) images were acquired using a 3D brain volume imaging (BRAVO) pulse sequence which uses an inversion recovery prepared fast spoiled gradient echo (FSPGR). The structural images were acquired using a 256 × 256 element matrix, 172 slices oriented to the anterior commissure- (AC-) posterior commissure (PC) plane, 1 mm^3^ isotropic voxels, 0.8 phase field of view factor, inversion time (TI) = 450 ms, TE = 3.224 ms, a flip angle of 13°, and an autocalibrated reconstruction for Cartesian sampling with a phase acceleration factor of 1.0 for the first session and 2.0 for all remaining sessions. All MRI procedures were conducted on a 3 Tesla (T) MRI (Discovery 750w, GE Healthcare, Madison, WI) using a 24-channel head coil.

Images of cerebral perfusion were acquired approximately 20 minutes prior to the application of tDCS and approximately 1.5 hours after the conclusion of stimulation using a pseudocontinuous arterial spin labeling (pcASL) technique [25] with inversion (tagging) pulses administered immediately inferior to the imaging volume. All images were acquired true axial (oriented perpendicular to the scanner bore) using a postlabel delay time (PLD) of 2025 ms. Five background suppression pulses were applied to reduce the signal of stationary tissues [26–28] and improve signal-to-noise ratio (SNR) of arterial blood. A 3D fast spin echo (3D FSE) sequence was used for acquisition of the imaging volume. To reduce motion sensitivity, improve acquisition time, and minimize susceptibility artifacts, a stack-of-spirals readout gradient starting at the center of the k-space was used [29]. A total of 8 spiral arms were used for k-space sampling. Echoes were rebinned to Cartesian space in a 128 × 128 matrix, with TR = 4640 ms, TE = 10.7 ms, voxel size = 1.875 × 1.875 mm, slice thickness = 4 mm, and flip angle = 111°. The sequence acquired a total of 3 tag/control pairs. The total acquisition time was 4 min 46 s. During the scan, participants were instructed to remain awake and focus on a fixation dot presented on the display. This condition has demonstrated significantly greater reliability in resting-state functional MRI across all within-network connections, as well as within default-mode, attention, and auditory networks when compared to eyes open (no specified fixation) and closed methods [30].

### 2.4. Data Processing and Analysis

Cerebral perfusion was quantified from ASL. CBF maps were computed from the automated functions in the GE reconstruction software. First, the 3 tagged and 3 control volumes were averaged in place (without motion correction). Then, difference images were calculated for all participants by subtracting the average tagged volume from the average control volume. Finally, quantitative CBF maps were generated from the difference images, the associated proton density- (PD-) weighted volumes, and a standard single compartment model [31–33].

**Figure 1:**
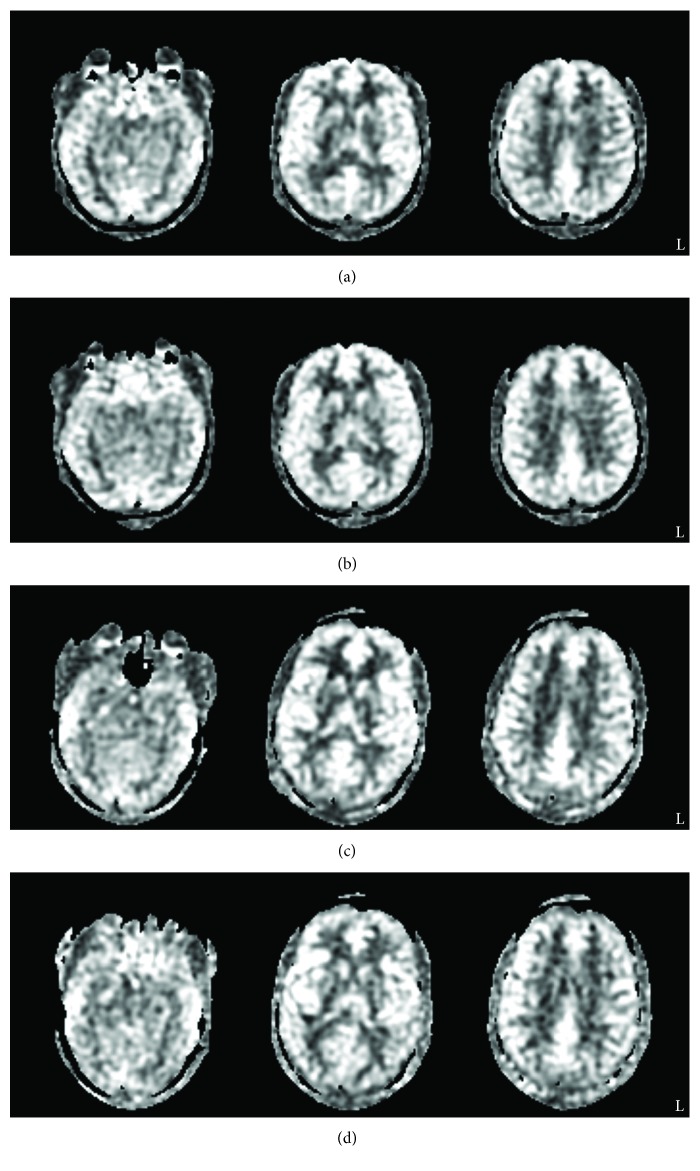
Raw CBF maps for (a) day 1 prestimulation and (b) day 3 poststimulation from a single CON participant and (c) day 1 prestimulation and (d) day 3 poststimulation from a single ACT participant.

The CBF maps from each day and session were exported from the MRI scanner and processed using the FMRIB Software Library (FSL) [34, 35] on a 74-core Rocks Cluster Distribution (http://www.rocksclusters.org) high-performance computing system capable of running 120 threads in parallel (see Figure 1, e.g., CBF maps). First, the PD-weighted images acquired were registered to the individual's high-resolution structural image by estimating motion from a boundary-based registration method which includes a fieldmap-based distortion correction [36]. Then, the individual's high-resolution structural image was registered to the MNI-152 T1-weighted 2 mm template provided in FSL [37, 38] using a 12-parameter model [39, 40]. In order to coregister all volumes, the CBF maps were converted to standard space using the transforms responsible for morphing the PD-weighted image of each data set to the structural image and the structural image to the template.

Next, group nonparametric statistical analyses were performed on the session 1 prestimulation and session 3 poststimulation coregistered CBF maps in a voxelwise fashion. Due to our mixed-model design and how the data would need to be permuted, an analysis of variance (ANOVA) was not possible using this approach. Instead, two separate analyses were performed. In the first, analyses were conducted separately for each group to evaluate the effect of the session. This analysis determined the statistical significance of differences in CBF (evaluated as increased perfusion from session 1 prestimulation to session 3 poststimulation) using permutation testing implemented in FSL's randomise [41, 42]. Null *t* distributions for contrasts representative of the main effect of the session were derived by performing 500,000 random permutations of the data [43]. A final *t* statistic was computed for each voxel by determining the probability of exceeding the *t* statistic from the known arrangement. Following this analysis, we implemented a clustering method to account for false positives due to the multiple comparisons [44]. This method considered adjacent voxels with a *t* statistic of 1.96 or greater to be a cluster. The significance of each cluster was estimated and compared to a threshold of *p* < 0.05 using Gaussian random field theory. The significance of voxels that either did not pass the significance level threshold or do not belong to a cluster was set to zero.

The second analysis assessed the interaction of the group and session using a single unpaired approach. Prior to this analysis, changes in CBF between the session 1 prestimulation and session 3 poststimulation coregistered CBF maps were calculated at the individual level. Then, the statistical significance of the variation in CBF between sessions and groups was determined using permutation testing implemented in FSL's randomise. Null *t* distributions for contrasts representative of the interaction of the session and group were derived by performing 500,000 random permutations. The clustering method outlined above was implemented to account for false positives due to multiple comparisons.

## 3. Results and Discussion

**Figure 2:**
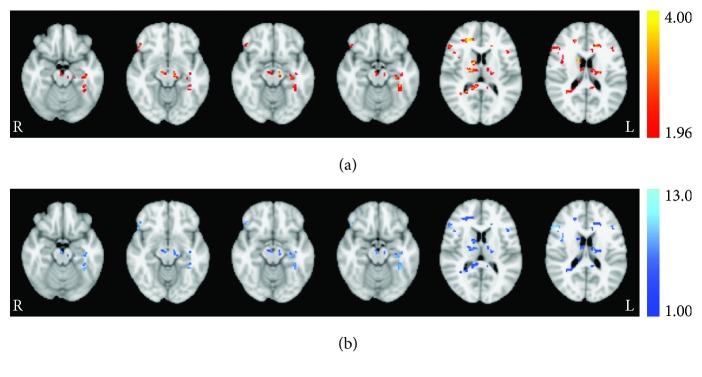
Session effect revealed from permutation testing for the ACT group. (a) Statistically significant (*t*-statistic) regions with altered CBF from baseline to session 3 poststimulation and (b) corresponding increases in quantified perfusion (mL/100 mg/min). Axial images taken at MNI coordinates *x* = −18, −12, −14, −16, 12, and 18 mm.

Paired permutation testing revealed a few small clusters with significant increases in resting CBF resulting from repetitive 2 mA stimulation of the left prefrontal cortex (Table 2, Figure 2). Localized increases in CBF were observed in several regions of the brainstem and cerebellum including the substantia nigra (SN). Cortically, bilateral changes in CBF were observed in the middle frontal, superior frontal, and inferior frontal gyri. Lateralized cortical changes were observed in the right rectal gyrus and precuneus and in the left supramarginal gyrus, paracentral lobule, parahippocampal gyrus, thalamus, caudate, and posterior cingulate cortex (PCC). However, the majority of the cortical effects appeared in white matter.

**Figure 3:**
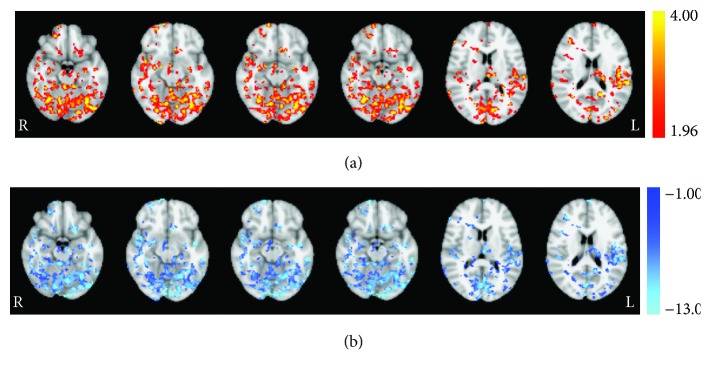
Session effect revealed from permutation testing for the CON group. (a) Statistically significant (*t*-statistic) regions with altered CBF from baseline to session 3 poststimulation and (b) corresponding decreases in quantified perfusion (mL/100 mg/min). Axial images taken at MNI coordinates *x* = −18, −12, −14, −16, 12, and 18 mm.

In contrast to the ACT group, paired permutation testing performed on the group receiving repetitive sham stimulation identified significant decreases in resting CBF (Table 3, Figure 3). This included a large cluster encompassing multiple subcortical brain regions. This also comprised of a bilateral decrease in the superior frontal gyrus. Furthermore, lateralized cortical decreases were observed in the right middle frontal gyrus, inferior frontal gyrus, precentral gyrus, superior temporal gyrus, thalamus, and putamen and in the left cuneus, precuneus, cingulate gyrus, fusiform gyrus, middle temporal gyrus, and medial frontal gyrus.

The unpaired permutation testing analyzed the difference in resting CBF from session 1 prestimulation to session 3 poststimulation between the ACT and CON groups. This analysis revealed an overall significantly larger decrease in resting CBF for the CON group (Tables 4 and 5). This included a large cluster encompassing multiple subcortical and cortical brain regions. This cluster is identified as the fusiform gyrus in Table 4 but also included projections beginning in the locus coeruleus (LC) and projecting through the SN and PCC. This also comprised of localized clusters across several cortical regions. CBF in the bilateral superior frontal gyrus was found to increase in the ACT group but decrease in the CON group. This effect also appeared in right-lateralized regions: inferior frontal and middle frontal gyri. Left-lateralized clusters in the medial frontal gyrus and fusiform and right-lateralized clusters in the precentral gyrus, thalamus, and putamen showed a significant decrease in perfusion in the CON group, but no significant changes were observed in the ACT group. The opposite was observed for the left inferior parietal lobule.

Systematic group variations in thickness or atrophy in gray matter and/or different gyrification patterns are plausible and may have resulted in some or all of the effects observed. To evaluate the possibility of anatomical variations between groups, we performed voxel-based morphometry (VBM) to investigate voxelwise differences in local gray matter volume and/or topography. This analysis utilized brain-extracted structural images to first produce a template. In order to not bias the template towards one group, 2 random subjects from the ACT group were not included in this step to ensure an equal number of samples represent each group. The brain-extracted images were segmented automatically into gray matter, affine-registered to the gray matter International Consortium for Brain Mapping (ICBM) 152 template [38], concatenated, and averaged. The average image was flipped along the *x*-axis, and the mirror images were reaveraged. The gray matter images were reregistered to the average template using nonlinear registration, concatenated, averaged, and flipped along the *x*-axis. A final symmetric gray matter template was created by averaging the mirror images from the nonlinear registration. Next, gray matter templates for all subjects were created and nonlinearly registered to the custom gray matter template. A compensation for gray matter variations due to the nonlinear transformation was introduced using the Jacobian of the warp field [45]. All the registered gray matter volumes were spatially smoothed using a Gaussian kernel (sigma = 4 mm). Finally, an unpaired *t*-test was performed to compare the gray matter volumes across groups using a permutation (number of permutations = 500,000) approach performed in FSL randomise. A threshold-free cluster enhancement method was utilized to correct for multiple comparisons. No significant findings were observed in this analysis indicating neither the thickness or atrophy in gray matter nor different gyrification patterns existed between groups. Furthermore, this suggests that these anatomical variations could not have caused the observed variations in perfusion.

The unpaired permutation analysis represents the interaction between the session and group and, thus, reveals the effects on cerebral perfusion attributable to the application of anodal left prefrontal tDCS. Cerebral perfusion measured from ASL is a correlate of metabolic processes [21]. In general, small, focal increases in perfusion were found in the group receiving 2 mA anodal left prefrontal tDCS across 3 consecutive days while a widespread decrease was observed in the group receiving sham stimulation. This implies metabolism was consistent in recurrent tDCS, and decreased metabolism is associated with sham stimulation.

Our study population was limited to active duty military members, and the study was executed in the evening after typical work days, although we did not measure or control sleep/wake times. After three consecutive days of study participation as outlined in Table 1, it is only reasonable that the participants would be experiencing symptoms of fatigue. Hypoperfusion measured from ASL has been observed and detailed in patients with chronic fatigue syndrome [46–48] and associated with cognitive fatigue in healthy individuals [49]. Furthermore, hypometabolism has been observed in patients with chronic fatigue syndrome [50] and multiple sclerosis with fatigue [51]. The results from our CON group are consistent with this postulation and these previous findings; however, the findings from our ACT group are not. We theorize that left prefrontal tDCS provides some neural mechanism that counteracts this neural effect of fatigue. Behaviorally, left prefrontal tDCS has been shown to reduce the cognitive decline associated with fatigue in a similar group of active duty military members in an extended wakefulness study [9]. Anodal tDCS applied to the motor cortex has also been shown to have behavioral effects from fatigue in patients with multiple sclerosis [52]. However, there are no studies to date that have evaluated the neural effects of tDCS on fatigue.

**Figure 4:**
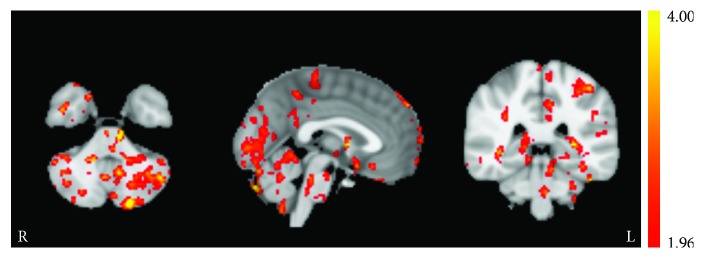
Session/group interaction effects (*t*-statistic) in the locus coeruleus revealed from permutation testing. The ACT group had significantly larger perfusion increases in the locus coeruleus from baseline to session 3 poststimulation compared to the CON group. The axial (left), sagittal (middle), and coronal (right) images were taken from MNI coordinates *z* = −40 mm, *y* = −36 mm, and *x* = 0 mm, respectively.

The altered perfusion observed in this work can be traced to the LC. The LC is well known as the largest noradrenergic nucleus in the brain. The noradrenergic system is responsible for the synthesis, storage, and release of norepinephrine. Although the LC is relatively small, it is the primary source of norepinephrine for the neocortex. Projections from the LC are diverse, innervating most of the central nervous system [53]. Norepinephrine is a neurotransmitter associated with increased arousal and alertness [46–48], enhances long-term and working memory processes [54], and promotes vigilance and sensory processing [55]. The evidence presented in this work suggests that repetitive 2 mA tDCS applied to the left prefrontal cortex sustains the metabolic activity of the LC (Figure 4) which may result in an increased production of norepinephrine and a decreased effect of fatigue. In this work, measurements of resting perfusion were collected approximately 1.5 hrs following the conclusion of stimulation. Therefore, this effect remains following stimulation; however, it is not known how long this effect persists. Previous sleep deprivation studies utilizing anodal left prefrontal tDCS observed single-session behavioral effects that persisted for many hours [9, 10]. Effects such as this and the current findings could be derived from activation of the noradrenergic system.

**Figure 5:**
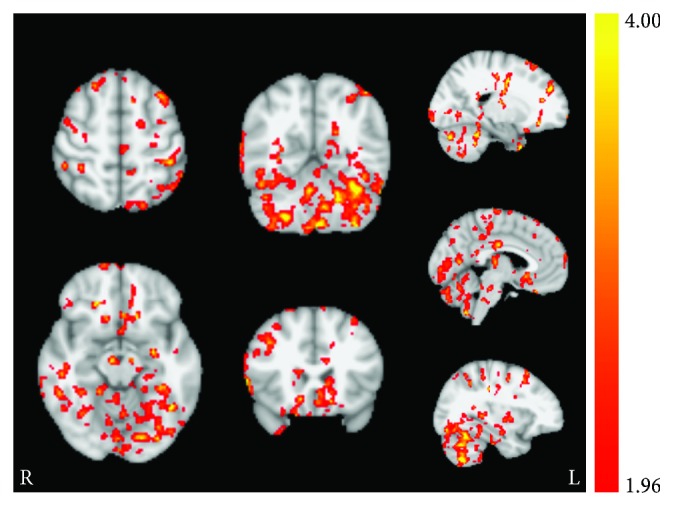
Session/group interaction effects (*t*-statistic) in the top-down attentional control network revealed from permutation testing. The ACT group had significantly larger perfusion increases in the right middle frontal gyrus, bilateral ACC, and left superior lobule from baseline to session 3 poststimulation compared to the CON group. The axial (left) images were taken from MNI coordinates *z* = −14 (top) and 56 (bottom) mm, sagittal (middle) images were taken from *y* = −58 (top) and 22 (bottom) mm, and coronal (right) images were taken from *x* = 35 (top), 47 (center), and 61 (bottom) mm.

Attention involves both top-down and bottom-up modulation. In bottom-up modulation, salient stimuli capture attention involuntarily while top-down modulation can direct attention as well as inhibit bottom-up processes. The ability to voluntarily direct attention (i.e., attentional control) varies significantly and substantially across individuals [56]. Top-down modulation of attention involves a variety of brain regions including the middle frontal gyrus, ACC, and superior parietal lobule. Each of these regions were found to have enhanced perfusion following repetitive 2 mA anodal tDCS to the left prefrontal cortex (Figure 5), suggestive of increased attentional control.

**Figure 6:**
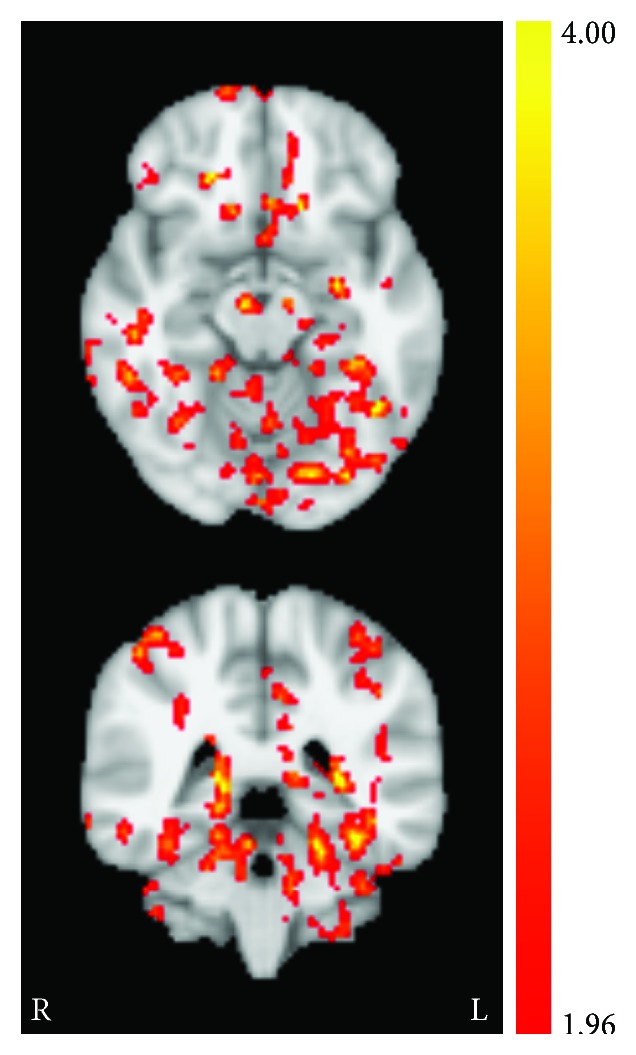
Session/group interaction effects (*t*-statistic) in the fusiform gyrus revealed from permutation testing. The ACT group had significantly larger perfusion increases in the right middle frontal gyrus, bilateral ACC, and left superior lobule from baseline to session 3 poststimulation compared to the CON group. The axial (top) and coronal (bottom) images were taken from MNI coordinates *z* = −14 mm and *y* = −40 mm, respectively.

Objects can be classified based upon the observation of physical properties such as shape, color, and texture. Semantic memory, general knowledge that has accumulated through life, can aid the classification process. The fusiform gyrus is theorized to largely contribute to processes involving semantic memory [57]. The large increase in perfusion in the occipital cortex, including the fusiform gyrus (Figure 6), is suggestive of enhanced utilization of semantic processes, increased semantic memory, and/or a heightened ability to recognize objects.

## 4. Conclusions

This study examined the effect of repetitive tDCS on cerebral perfusion. Anodal left prefrontal tDCS was used to apply 2 mA to the scalp for 30 minutes on three consecutive days. Measures of resting cerebral perfusion were acquired before and after stimulation on each day using ASL. Widespread increases in perfusion, indicative of increased metabolism, were observed; however, general decreases were observed in a matched group receiving sham tDCS. Furthermore, perfusion increased significantly more in the active stimulation group across many areas of the brain. These increases originated in the LC and spread extensively to regions in the neocortex supporting functions such as object recognition and top-down attentional modulation. The changes in the neocortex may be a direct result of the stimulation or an indirect result via the changes in the noradrenergic system produced from the altered LC activity. These findings help understand the broad behavioral effects that have been demonstrated using anodal left prefrontal tDCS. Future work is necessary to identify if the observed changes in perfusion correlate with altered metabolism but should also address the transiency of these effects.

## Figures and Tables

**Table 1:** Starting times for the experimental procedures. Participants completed the three sessions in groups of two with staggered start times.

Procedure	Participant 1			Participant 2		
	Local start time	ASL scan time	Local end time	Local start time	ASL scan time	Local end time
Prestimulation MRI	5:00 pm	6:10 pm	6:15 pm	6:15 pm	7:25 pm	7:30 pm
Transcranial DC stimulation	6:30 pm	n/a	7:00 pm	7:55 pm	n/a	8:25 pm
Poststimulation MRI	7:30 pm	8:40 pm	8:45 pm	8:45 pm	9:55 pm	10:00 pm

**Table 2:** The largest 15 clusters identified with an average increase in perfusion between prestimulation at session 1 and poststimulation at session 3 (ΔCBF) for the ACT group.

Volume (mm^3^)	Max *t*-statistic	Max ΔCBF (mL/100 mg/min)	Max ΔCBF location (mm)		
			*X*	*Y*	*Z*
8000	7.23	13.45	−4	16	64
3824	5.28	15.00	16	−52	34
3320	3.76	10.27	−36	−60	52
3136	4.61	15.73	50	14	28
2832	4.66	8.64	−8	−12	10
2632	5.39	9.09	6	−12	12
2312	4.21	9.45	29	−40	38
2280	3.68	17.82	0	−28	44
1984	4.87	11.73	42	12	34
1800	5.00	13.09	−34	−26	−22
1528	6.58	10.09	40	32	4
1048	3.57	12.00	40	−72	−52
1032	3.79	9.18	−22	−36	10
864	6.59	13.73	0	−44	28
768	3.07	14.00	−6	−48	−52

**Table 3:** The largest 15 clusters identified with an average decrease in perfusion between prestimulation at session 1 and poststimulation at session 3 (ΔCBF) for the CON group.

Volume (mm^3^)	Max *t*-statistic	Max ΔCBF (mL/100 mg/min)	Max ΔCBF location (mm)		
			*X*	*Y*	*Z*
204,208	11.3	−23.00	−4	76	64
4016	7.41	−12.78	16	−20	34
3528	4.83	−24.56	−36	−60	52
2912	5.37	−12.67	50	14	28
2560	3.91	−13.11	−8	−12	10
1976	8.95	−13.89	6	−12	12
1344	4.34	−12.22	32	−40	38
1336	5.01	−9.44	0	−28	44
1296	5.84	−9.56	42	12	34
1112	4.75	−15.33	−34	−26	−22
944	5.48	−13.00	40	32	4
840	5.35	−20.44	40	−72	−52
832	4.62	−9.89	−22	−36	10
824	7.72	−22.11	0	−44	28
816	4.38	−9.89	−16	−42	30

**Table 4:** Clusters identified with a significantly larger increase in perfusion from prestimulation at session 1 to session 3 poststimulation for the ACT group than for the CON group.

Hemisphere	Lobe	Gyrus	Volume (mm^3^)	Max *t*-statistic	Max *t*-statistic location (mm)		
					*X*	*Y*	*Z*
Left	Temporal	Fusiform gyrus	15,784	5.14	−44	−56	−12
Right	Frontal	Inferior frontal gyrus	1445	5.66	60	20	4
Left	Parietal	Inferior parietal lobule	981	4.09	−32	−28	38
Left	Frontal	Superior frontal gyrus	715	4.09	−16	50	−20
Right	Parietal	Inferior parietal lobule	454	4.13	38	12	26
Left	Parietal	Superior parietal lobule	412	3.96	−44	−58	58
Left	Frontal	Precentral gyrus	293	4.43	−36	2	24
Right	Temporal	Fusiform gyrus	252	3.87	50	−32	−28
Right	Temporal	Middle temporal gyrus	210	3.93	42	0	−26
Right	Temporal	Inferior temporal gyrus	208	5.34	68	−30	−18
Right	Limbic	Cingulate	206	4.82	20	−4	42
Right	Parietal	Postcentral gyrus	177	3.24	62	−12	24
Right	Frontal	Precentral gyrus	176	3.08	50	2	48
Right	Limbic	Anterior cingulate cortex	166	3.67	12	22	−12
Left	Frontal	Medial frontal gyrus	148	3.42	−12	2	60
Left	Frontal	Superior frontal gyrus	131	3.93	−6	32	62
Right	Frontal	Superior frontal gyrus	101	3.78	18	70	−6
Right		Thalamus	95	3.64	8	−10	18
Right	Frontal	Middle frontal gyrus	87	3.37	22	20	64
Right	Parietal	Postcentral gyrus	86	3.54	44	−20	46
Left	Limbic	Anterior cingulate cortex	82	3.35	−14	30	22
Right	Limbic	Cingulate	80	3.53	24	−18	44
Left	Frontal	Medial frontal	80	3.28	−8	30	40
Left	Frontal	Superior frontal gyrus	77	2.91	−2	68	18
Left	Temporal	Fusiform gyrus	73	4.27	−46	−12	−28
Left	Frontal	Precentral gyrus	73	3.35	−38	−6	46
Right	Occipital	Lingual gyrus	71	3.45	26	−102	−6
Right		Putamen	71	3.09	−32	−8	−2

**Table 5:** Average CBF (±SEM) for each session/group from the clusters identified with a significantly larger increase in perfusion from prestimulation at session 1 to session 3 poststimulation for the ACT group than for the CON group.

Cluster	ACT		CON	
	Day 1 prestimulation CBF (mg/100 mL/min)	Day 3 poststimulation CBF (mg/100 mL/min)	Day 1 prestimulation CBF (mg/100 mL/min)	Day 3 poststimulation CBF (mg/100 mL/min)
L. fusiform gyr.	46.05 ± 1.67	49.12 ± 2.72	54.32 ± 2.95	44.92 ± 3.11
R. inf. front. gyr.	45.66 ± 1.74	51.35 ± 2.66	55.39 ± 2.14	48.77 ± 1.80
L. inf. par. lob.	41.91 ± 2.33	46.66 ± 2.85	53.84 ± 2.97	48.20 ± 2.31
L. sup. front. gyr.	43.85 ± 1.66	49.38 ± 2.28	54.18 ± 3.77	45.59 ± 2.08
R. inf. par. lob.	54.02 ± 2.71	60.72 ± 3.61	62.01 ± 2.61	56.53 ± 2.17
L. sup. par. lob.	46.36 ± 2.06	51.62 ± 2.46	57.50 ± 3.72	51.70 ± 2.37
L. precentral gyr.	47.67 ± 2.56	54.85 ± 3.32	60.10 ± 3.59	53.57 ± 2.24
R. fusiform gyr.	45.13 ± 1.75	49.94 ± 2.78	54.71 ± 2.83	47.77 ± 2.10
R. mid. temp. gyr.	35.97 ± 1.72	38.91 ± 2.66	44.12 ± 3.19	36.85 ± 2.29
R. inf. temp. gyr.	54.20 ± 2.66	60.18 ± 3.50	67.23 ± 3.00	59.48 ± 3.20
R. cingulate	26.44 ± 1.62	29.40 ± 1.67	31.46 ± 1.57	26.75 ± 1.24
R. postcentral gyr.	55.16 ± 2.92	58.52 ± 3.74	66.35 ± 2.57	58.83 ± 3.34
R. precentral gyr.	50.97 ± 2.50	55.60 ± 3.57	59.40 ± 2.75	52.78 ± 2.22
R. ant. cing. cort.	43.86 ± 2.98	47.93 ± 3.31	50.55 ± 1.89	43.44 ± 2.40
L. med. front. gyr.	38.35 ± 2.21	44.37 ± 2.64	50.01 ± 2.87	44.60 ± 1.31
L. sup. front. gyr.	45.39 ± 2.39	51.99 ± 1.73	58.87 ± 3.47	52.62 ± 2.46
R. sup. front. gyr.	55.52 ± 1.68	61.90 ± 3.42	70.82 ± 5.29	59.93 ± 2.77
R. thalamus	37.73 ± 2.26	43.27 ± 2.47	42.38 ± 1.91	39.36 ± 2.03
R. mid. front. gyr.	47.86 ± 3.26	54.08 ± 3.30	60.84 ± 2.89	52.83 ± 2.26
R. postcentral gyr.	44.32 ± 1.83	49.13 ± 2.65	51.83 ± 3.68	45.45 ± 3.21
L. ant. cing. cort.	26.56 ± 1.71	31.58 ± 1.28	38.40 ± 4.57	31.94 ± 1.78
R. cingulate	45.18 ± 3.25	49.41 ± 3.54	54.01 ± 2.76	46.79 ± 1.87
L. med. front. gyr.	21.89 ± 1.24	26.22 ± 1.71	27.54 ± 1.55	24.80 ± 1.47
L. sup. front. gyr.	48.97 ± 1.86	54.11 ± 4.37	60.99 ± 5.80	51.25 ± 3.37
L. fusiform gyr.	43.57 ± 2.65	49.91 ± 3.64	58.01 ± 2.42	52.00 ± 2.71
L. precentral gyr.	37.09 ± 1.91	40.43 ± 2.50	47.73 ± 3.50	39.29 ± 2.63
R. lingual gyr.	39.00 ± 1.94	41.87 ± 1.60	46.74 ± 3.45	40.15 ± 2.49
R. putamen	47.96 ± 3.36	48.57 ± 2.53	56.82 ± 4.07	46.27 ± 3.81

## Data Availability

This project is funded by a DoD contract, and the data is not available for public release at this time.
